# Salvianolic Acid B Attenuates Rat Hepatic Fibrosis via Downregulating Angiotensin II Signaling

**DOI:** 10.1155/2012/160726

**Published:** 2012-11-12

**Authors:** Shu Li, Lina Wang, Xiuchuan Yan, Qinglan Wang, Yanyan Tao, Junxia Li, Yuan Peng, Ping Liu, Chenghai Liu

**Affiliations:** ^1^Institute of Liver Diseases, ShuGuang Hospital, Shanghai University of Traditional Chinese Medicine, Shanghai 201203, China; ^2^Department of Traditional Chinese Medicine, Changhai Hospital, Second Military Medical University, Shanghai 200433, China; ^3^Shanghai Key Laboratory of Traditional Chinese Clinic Medicine, Shanghai 201203, China; ^4^E-Institute of TCM Internal Medicine, Shanghai Municipal Education Commission, Shanghai 201203, China

## Abstract

The renin-angiotensin system (RAS) plays an important role in hepatic fibrosis. Salvianolic acid B (Sal B), one of the water-soluble components from Radix Salviae miltiorrhizae, has been used to treat hepatic fibrosis, but it is still not clear whether the effect of Sal B is related to angiotensin II (Ang II) signaling pathway. In the present study, we studied Sal B effect on rat liver fibrosis and Ang-II related signaling mediators in dimethylnitrosamine-(DMN-) induced rat fibrotic model *in vivo* and Ang-II stimulated hepatic stellate cells (HSCs) *in vitro*, with perindopril or losartan as control drug, respectively. The results showed that Sal B and perindopril inhibited rat hepatic fibrosis and reduced expression of Ang II receptor type 1 (AT1R) and ERK activation in fibrotic liver. Sal B and losartan also inhibited Ang II-stimulated HSC activation including cell proliferation and expression of type I collagen I (Col-I) and **α**-smooth muscle actin (*α*-SMA) production *in vitro*, reduced the gene expression of transforming growth factor beta (TGF-*β*), and downregulated AT1R expression and ERK and c-Jun phosphorylation. In conclusion, our results indicate that Sal B may exert an antihepatic fibrosis effect via downregulating Ang II signaling in HSC activation.

## 1. Introduction

Hepatic fibrosis is a common response to most chronic liver diseases, which may lead to cirrhosis, and cause liver failure as well as increase the risk for hepatocellular carcinoma. However, there is currently no approved effective therapy to reverse the progression of hepatic fibrosis. Hepatic stellate cells (HSC), the predominant cell type in the liver, play a crucial role in the process of hepatic fibrosis [1]. Once HSC becomes activated, it will promote the production of extracellular matrix (ECM) but decrease ECM degradation, which eventually leads to the abnormal deposition of ECM and hepatic fibrosis [2, 3]. 

In recent years, there is much evidence to support that the renin-angiotensin system (RAS) plays an important role in HSC activation and hepatic fibrogenesis [4–7]. Angiotensin II (Ang II), the main effector of RAS, is frequently elevated in patients' serum with cirrhosis, the hepatic RAS is also upregulated in both human and rat livers undergoing active fibrogenesis [8, 9]. Infusion of Ang II induces oxidative stress, hepatic inflammation, and vascular damage, resulting in liver injury in rats [10]. In addition, patients with chronic hepatitis C and a genetic polymorphism that results in increased synthesis of Ang II develop more severe fibrosis [11]. Ang II regulates HSC activation and hepatic fibrosis through its signaling pathway including Ang II receptor type 1 (AT1R) and its downstream mediators [12–14]. Activation of AT1R by Ang II induces contraction and proliferation of HSCs [7, 15] and increases ECM proteins expression [4, 16]. Moreover, Ang II can exert proinflammatory and profibrogenic actions by increasing the secretion of cytokines, especially transforming growth factor beta 1 (TGF-*β*1) [17]. Ang-II intracellular signaling pathway involves in oxidative stress, transcription factors nuclear factor *κ*B (NF-*κ*B), and activating protein-1 (AP-1), in particular signaling molecules called mitogen-activated protein kinase (MAPK) [10, 18]. Many studies have shown that inhibition of Ang II or blockade of AT1R reduces the HSC activation and collagen synthesis and attenuates hepatic fibrosis [19–22]. AT1R-deficient mice also exhibit attenuated response to hepatic inflammation and fibrosis [13, 23]. Thus the inhibition of Ang II signaling could potentially abrogate hepatic fibrosis [24]. 

There is a continuing interest in the treatment of hepatic fibrosis with traditional Chinese medicine (TCM) in our institute. We previously developed a formula with antihepatic fibrosis activity, Fuzheng Huayu recipe (FZHY), which now was widely used as AN antiliver fibrotic product in China [25–28]. Later we found that Radix Salviae miltiorrhizae (Danshen) is the main effective herb in FZHY, and salvianolic acid B (Sal B), one of the water soluble components from Radix Salviae miltiorrhizae, has good action against hepatic fibrosis in animal model and patients with chronic hepatitis B [29, 30]. However, the action mechanisms of Sal B on liver fibrosis have not been thoroughly elucidated, especially those related to Ang II signaling pathway. In view of the importance of RAS in liver fibrogenesis, we performed the current experiments to elucidate the effects of Sal B on Ang II signaling in HSC activation and thereby understand the action mechanisms of Sal B and FZHY against liver fibrosis. 

## 2. Materials and Methods 

### 2.1. Chemicals and Drugs

Sal B was a generous gift from Professor Da-yuan Zhu at the Shanghai Institute of Meteria Medica, Chinese Academy of Science, Shanghai, China. The purity of Sal B was ≥98%. Its molecular weight is 718, and its molecular formula is C_36_H_30_O_16_. Sal B was dissolved in DMSO at a concentration of 100 mM and stored at −20°C. Ang II was purchased from Sigma-Aldrich (St. Louis, MO, USA). Antibodies against AT1 (SantaCruz, sc-1173), pERK1/2 (SantaCruz, sc-7383), total ERK2 (SantaCruz, sc-1467), GAPDH (SantaCruz, sc-166574), Col-I (Sigma-Aldrich, c2456), alpha-smooth muscle actin (*α*-SMA) (Abcam, ab5649), *β*-tubulin (Cell Signaling Technology, 2146), c-Jun (Cell Signaling Technology, 9165), and p-c-Jun (Cell Signaling Technology, 9261) were used in this study. 

### 2.2. Cell Culture

HSC-T6 cells were cultured in DMEM supplemented with 10% fetal bovine serum (FBS), 100 units/mL of penicillin, and 100 units/mL of streptomycin. The cells were cultured with 5% CO_2_ at 95% humidity. 

### 2.3. HSC Isolation and Culture

Primary HSCs were isolated from normal rat liver by perfusion with pronase and followed by collagenase and Nycodenz density-gradient centrifugation method [31]. Primary HSC was cultured with DMEM supplemented with 20% FBS. Primary HSC was seeded in 6-, 12-, and 96-well plates at the 5th day. The experiments were performed in accordance with the Helsinki Declaration of 1975.

### 2.4. MTT Assay

To determine the cell proliferation, HSC was seeded at a density of 5,000 cells per well in 96-well cell culture plates. After growing for 24 h under normal growth conditions, the medium was replaced with DMEM containing Ang II (10–6 M) and different concentrations of Sal B. The cells were incubated for the indicated times. Then MTT (5 mg/mL) was added to every well and plateswere incubatedfor 4 h. DMSO wasaddedand the absorbance at 490 nm was read using a microplate reader. 

### 2.5. Immunofluorescence

Cells were plated on glass coverslips (Fisher Scientific) in 12-well culture dishes. After growing for 24 h under normal growth conditions, the cells were starved in DMEM containing 0.5% FBS and pretreated with Sal B for 4 h followed by Ang II (10–6 M) incubation for another 24 h. Cells were then washed twice with cold serum-free media (DMEM) and then fixed in 4% paraformaldehyde in PBS for 10 minutes. Immunofluorescence was performed to detect the F-actin and *α*-SMA as described previously [32]. Images were taken using Cellomics ArrayScan VTI HCS Reader and analyzed using Cellomics Cell Health Profiling BioApplication Software.

### 2.6. Animals

Wistar male rats were obtained from the Shanghai Laboratory Animal Center, Chinese Academy of Sciences, and maintained in a room under a temperature controlled at 23 ± 2°C and a 12 h light-dark lighting cycle. The animals were allowed a standard pellet chow and water ad libitum. 

The rats were randomly divided into 4 groups: normal (*n* = 6), model (*n* = 10), Sal B Treatment (Sal B) (*n* = 10), and perindopril treatment (PER) (*n* = 10). The liver interstitial fibrosis model was induced by intraperitoneal (i.p) injection of dimethylnitrosamine (DMN) at a dose of 10 *μ*g/kg body weight once every other day for 4 weeks. From the third week of DMN injection, rats in the Sal B and PER groups were treated with Sal B at a dose of 10 mg/kg body weight and perindopril at a dose of 5 mg/kg body weight, respectively, once a day for 4 weeks. Rats in normal and model groups were treated with equal amount of vehicle. Rats were sacrificed after treatments and the livers were removed. A portion of each liver was fixed in 10% phosphate-buffered formalin for histological studies after paraffin embedding. The remainder was snap-frozen in liquid nitrogen and stored at −80°C for western blot. The experiments were performed in accordance with the Helsinki Declaration of 1975 and approved by the Ethics Committee of ShuguangHospital. All the animals used in the study received humane care.

### 2.7. Sirius Red Staining

The left lateral lobe of the liver was sliced, and tissue slices were fixed in 10% buffered-neutral formalin for 24 h. The fixed liver tissue slices were embedded in paraffin, sectioned, deparaffinized, and rehydrated using standard techniques. Sections 5 *μ*m in thickness were subjected to Sirius red staining as described previously [30]. An arbitrary scope was given to each microscopic field viewed at a magnification of 200x. 

### 2.8. Western Blot

HSC was lysed with RIPA (150 mM NaCl, 1% Nonidet P-40, 0.1% SDS, 50 mM Tris-HCl pH 7.4, 1 mM EDTA, 1 mM PMSF, 1x Roche complete mini protease inhibitor cocktail, Roche PhosSTOP phosphatase inhibitor cocktail). The supernatants were collected after centrifugation at 10,000 g at 4°C for 15 min. Protein concentration was determined using a BCA protein assay kit (Thermo Scientific, Shanghai, China). Equal amounts of protein were separated by 10% SDS gel electrophoresis (SDS-PAGE) under denaturing and nonreducing conditions and then transferred to a nitrocellulose membrane. The membrane was blocked with 5% nonfat milk in TBST at room temperature for 1 h and then incubated with primary antibody at 4°C overnight. After washing with TBST, the blots were incubated with horseradish-coupled secondary antibody. The signals were visualized using the OdysseyImagingSystem (LI-COR Bioscience). Since the primary anti-ERK2 and primary anti-GAPDH could employ the same secondary antibody, ERK2 and GAPDH were exposed on the same membrane.

### 2.9. RNA Isolation, cDNA Synthesis, and Real-Time RT-PCR

Total RNA was isolated using Trizol Reagent (Invitrogen, Shanghai, China) according to the manufacturer's protocol. RNA concentration was determined by a UV spectrophotometer. cDNA was generated using 1 *μ*g of total RNA in a final reaction volume of 20 *μ*L by the first-strand cDNA synthesis kit (TOYOBO, Osaka, Japan) according to the manufacturer's protocol. Quantitative real-time PCR was performed with an ABI stepone plus real-time PCR system. Primers along with their sequences are listed in Table 1. PCR mixtures contained 2 *μ*L of cDNA, 10 *μ*L of SYBR Premix 2x and 0.25 *μ*mol/L of forward and reverse primers, for a total volume of 20 *μ*L. Reactions were started with a polymerase activation step at 94°C for 10 min followed by 40 cycles of 94°C for 10 s, 60°C for 20 s, and 72°C for 25 s. Fluorescent data were acquired after each cycle. The absence of nonspecific products was verified after each run by melting curve analysis. The relative gene quantities were calculated by 2^−ΔΔCT^ method.

### 2.10. Statistics

Data are expressed as mean ± standard deviation. Data were analyzed using a one-way analysis of variance as well as the least significant difference test, and *P* < 0.05 was considered statistically significant.

## 3. Results

### 3.1. Sal B Inhibited DMN-Induced Rat Liver Fibrosis

**Figure 1:**
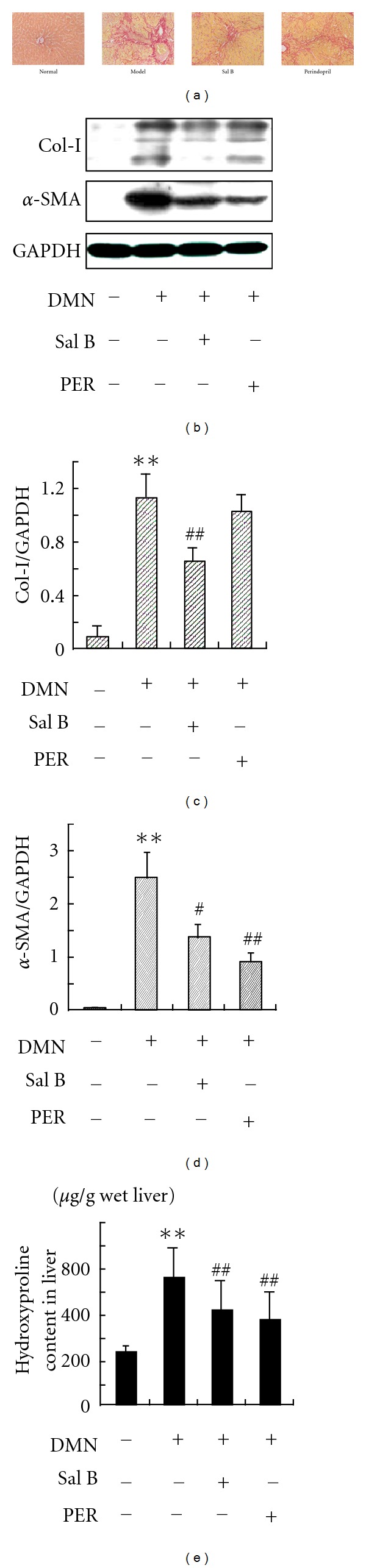
Effects of Sal B and perindopril (PER) on DMN-induced rat hepatic fibrosis. (a) Representative photomicrographs of Sirius red staining for rat liver. (b) western blot analysis of Col-I in rat liver. Rat liver protein was extracted and the samples were electrophoresed and analyzed by Western blot with Col-I antibody. ((c) and (d)) Quantities for densitometric analysis of Col-I and *α*-SMA. Each bar represents the means ± S.D. of three rats assayed in triplicate. (e) Hydroxyproline content in normal and DMN-induced rats livers. **P < 0.01 versus normal; ^##^ *P* < 0.01 versus model.

Many studies in our institute have demonstrated that Sal B could inhibit liver fibrosis. To confirm the inhibitory effects of Sal B on liver fibrosis induced by DMN, liver fibrosis and Col-I expression was determined by Sirius red staining (Figure 1(a)) and western blot (Figures 1(b) and 1(c)). The normal group rats showed normal architecture. Expression of Col-I in DMN-treated group rats increased significantly compared with that of control group rats. As shown by Sirius red staining, treatment with Sal B caused resolution of fibrosis, although deposition of ECM was observed in the liver. Although the Col-I level in perindopril group had a trend of decreasing, no significant difference was detected. The *α*-SMA expression induced by DMN was significantly inhibited by Sal B and also by perindopril (Figure 1(d)). In DMN-treated rats, hydroxyproline content was also elevated compared to that of normal rats (Figure 1(e)). Treatment with Sal B or perindopril downregulated the hydroxyproline content significantly.

### 3.2. Sal B Downregulated AT1R and ERK Phosphorylation in Rat Liver

**Figure 2:**
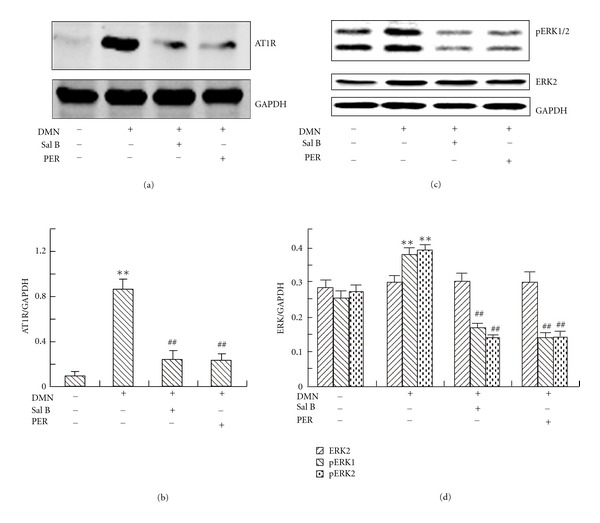
Effects of Sal B and perindopril (PER) on AT1R and ERK expression in rat liver. Protein was extracted from rat liver and the samples were electrophoresed and analyzed by western blot with AT1R (a) or ERK/pERK (b) antibodies. ((c) and (d)): Quantities for densitometric analysis of AT1R and ERK/pERK. Each bar represents the means ± S.D. of three rats assayed in triplicate. **P < 0.01 versus normal; ^##^ *P* < 0.01 versus model.

To further elucidate the mechanisms of Sal B effects, we first determined the expression of AT1R in rat liver by western blot. In normal rats, there was little expression of AT1R (Figure 2(a)). DMN administration could significantly increase the AT1R level. However, perindopril and Sal B treatment reduced the AT1R expression. DMN also induced ERK phosphorylation in rat liver (Figure 2(b)), which is a crucial step for Ang II's effect. The phosphorylation of ERK induced was significantly suppressed by Sal B and perindopril, but there was little effect of Sal B and perindopril on total ERK expression.

### 3.3. Sal B Inhibited Ang II-Induced HSC Proliferation

**Figure 3:**
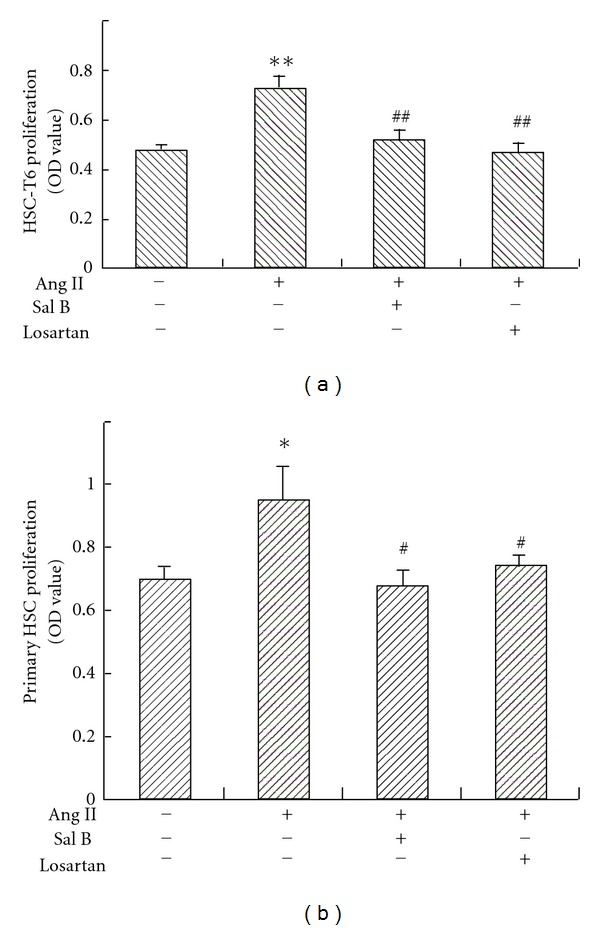
Effects of Sal B and losartan on Ang II-induced HSC proliferation. (a) HSC-T6 or (b) primary HSC cells were seeded in 96-well plates in DMEM medium with 10% or 20% FBS, respectively. After 24 h, cells were pretreated with Sal B (10^−5^ M), losartan (10^−6^ M), or vehicle in DMEM medium with 0.5% FBS for 4 h, and then incubated with Ang II (10^−6^ M) for another 24 h. Cells proliferation was determined by MTT method. Each bar represents the mean ± S.D. **P < 0.01 versus normal; ^##^ *P* < 0.01 versus Ang II alone group.

In accordance with previous reports [7, 33, 34], Ang II stimulated HSC proliferation in the present study. Sal B and losartan inhibited both the HSC-T6 and primary HSC proliferation induced by Ang II (Figures 3(a) and 3(b)). 

### 3.4. Sal B Inhibited Ang II-Induced *α*-SMA and Col-I Expression in HSC

**Figure 4:**
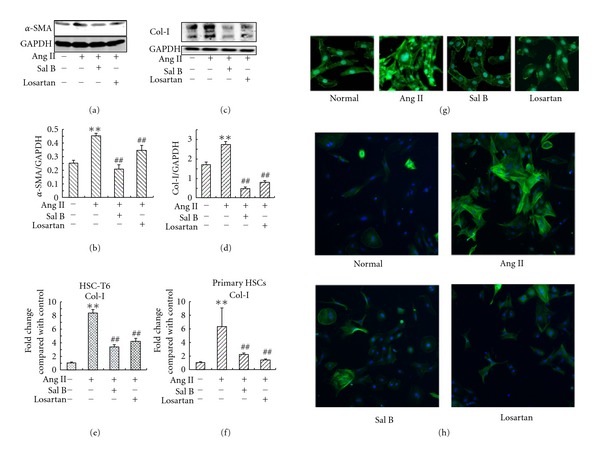
Effects of Sal B and losartan on Ang II-induced *α*-SMA and Col-I expression in HSC. HSC-T6 cells or primary rat HSC were seeded in 6 cm dishes in DMEM medium with 10% FBS. After 24 h, cells were pretreated with Sal B (10^−5^ M), losartan (10^−6^ M), or vehicle in DMEM medium with 0.5% FBS for 4 h, and then incubated with Ang II (10^−6^ M) for another 24 h. Western blot and real-time RT-PCR were performed to detect *α*-SMA and Col-I level. (a) *α*-SMA expression in HSC-T6 cells; (b) quantities for densitometric analysis of *α*-SMA; (c) Col-I expression in HSC-T6 cells; (d) quantities for densitometric analysis of Col-I; (e) Col-I mRNA levels in HSC-T6 cells; (f) Col-I mRNA levels in primary rat HSC; (g) staining for F-actin in HSC-T6 cells; (h) staining for *α*-SMA expression in primary rat HSC. Each bar represents the mean ± S.D. of three independent experiments. **P < 0.01 versus normal; ^##^ *P* < 0.01 versus Ang II alone group.

Ang II is able to induce the activation of HSC and overproduction of ECM proteins in HSC [34]. To observe the effect of Sal B on Ang II-induced HSC activation and ECM expression, we performed western blot and real-time RT-PCR to examine the *α*-SMA and Col-I levels. Treatment with Ang II obviously upregulated the expression of *α*-SMA (Figures 4(a) and 4(b)) and Col-I (Figures 4(c) and 4(d)) in HSC-T6 cells. Pretreatment with Sal B or losartan for 4 h notably reduced Ang II-induced Col-I and *α*-SMA secretion. In primary rat HSC, Ang II-induced overexpression of *α*-SMA was also significantly reduced by Sal B and losartan as shown in Figure 4(h). Col-I mRNA elevated about 8-fold after Ang II stimulation in HSC-T6 cells. Both Sal B and losartan could decrease the Col-I mRNA expression (Figure 4(e)). In primary rat HSC, similar result was obtained (Figure 4(f)). In Ang II treated HSC (Figure 4(g)), prominent F-actin reorganization was observed by immunofluorescence. Staining for F-actin demonstrated significant loss of F-actin in response to Sal B and losartan. 

### 3.5. Sal B Inhibited Ang II-Induced TGF-*β* mRNA Upregulation

**Figure 5:**
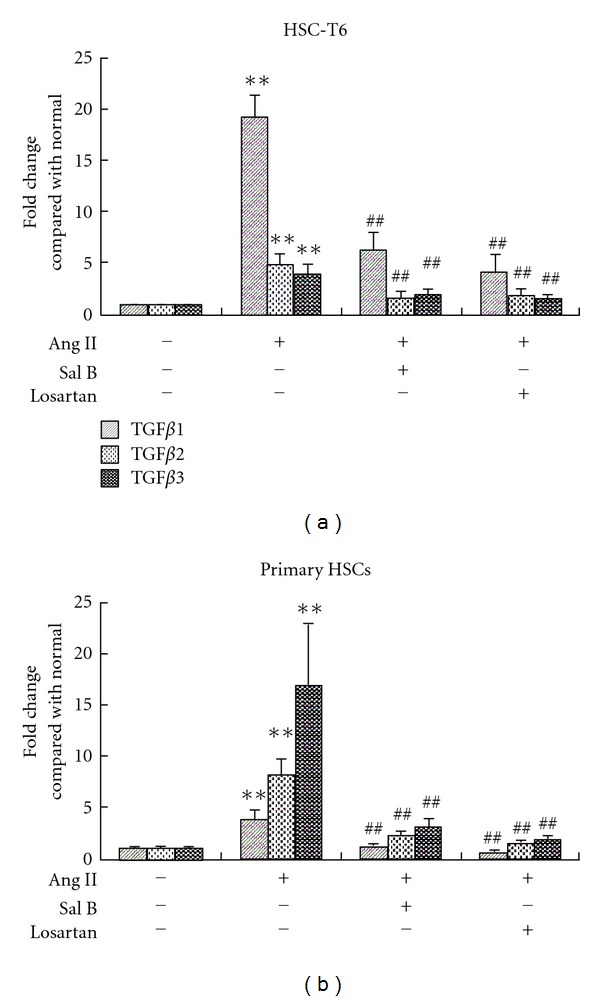
Effects of Sal B and losartan on Ang II-induced TGF-*β* expression in HSC. HSC-T6 cells or primary rat HSC was seeded in 12-well plate in DMEM medium with 10% FBS. After 24 h, cells were pretreated with Sal B (10^−5^ M), losartan (10^−6^ M), or vehicle in DMEM medium with 0.5% FBS for 4 h, and then incubated with Ang II (10^−6^ M) for another 24 h. real-time RT-PCR was performed to examine mRNA levels of all three TGF-*β* isoforms in (a) HSC-T6 cells and (b) primary rat HSC. Relative expression values are shown asfold changes compared with normal. Each bar represents the mean ± S.D. of three independent experiments. **P < 0.01 versus normal; ^##^ *P* < 0.01 versus Ang II alone group.

Since TGF-*β* plays a central role in the activation of HSC after stimulation of Ang II. Therefore, we studied the production of TGF-*β* mRNA levels in HSC-T6 cells and primary rat HSC. HSC was incubated with Ang II in the presence or absence of Sal B or losartan for 24 h. Stimulation of HSC with Ang II caused a significant increase of mRNA levels of all three TGF-*β* isoforms in HSC (Figure 5). Both Sal B and losartan reduced the TGF-*β* mRNA up-regulation induced by Ang II in HSCs.

### 3.6. Sal B Inhibited Ang II-Induced ERK and c-Jun Activation in HSC

**Figure 6:**
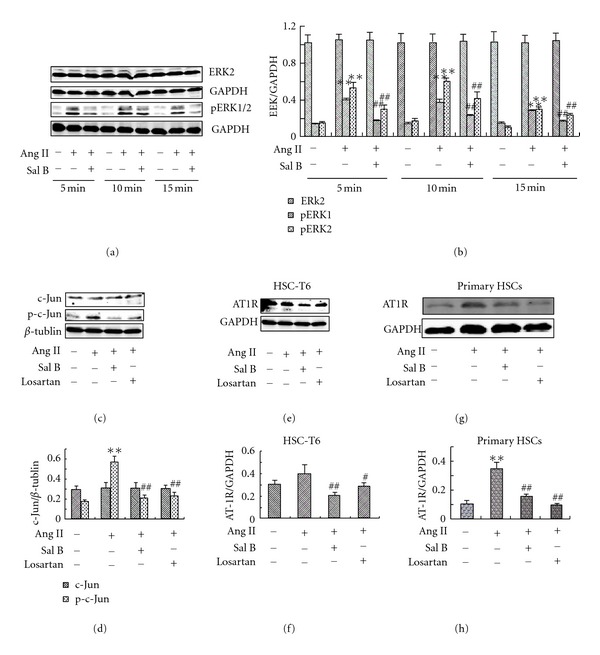
Effects of Sal B and losartan on Ang II-induced ERK and c-Jun phosphorylation and AT1R expression in HSC. HSC-T6 cells or primary rat HSC were seeded in 6 cm dishes in DMEM medium with 10% FBS. After 24 h, cells were pretreated with Sal B (10^−5^ M), losartan (10^−6^ M) or vehicle in DMEM medium with 0.5% FBS for 4 h, then incubated with Ang II (10^−6^ M) for indicated times. (a) Western blot analysis of total and phosphorylated ERK in HSC-T6 cells; (b) quantities for densitometric analysis of ERK and pERK; (c) western blot analysis of c-Jun and p-c-Jun in HSC-T6 cells; (d) quantities for densitometric analysis of c-Jun and p-c-Jun; (e) and (g) western blot analysis of AT1R protein level in HSC-T6 cells and primary rat HSC, respectively; (f) and (h) quantities for densitometric analysis of AT1R in HSC-T6 cells and primary rat HSC, respectively. Each bar represents the mean ± S.D. of three independent experiments. **P < 0.01 versus normal; ^##^ *P* < 0.01 versus Ang II alone group.

Activation of ERK and c-Jun are important steps for HSC to produce ECM in Ang II related pathway. Decreased phosphorylation of c-Jun and p42/44 MAPK were shown in AT1knockout mice compared to AT1wild type animals [13]. In the present study, Ang II induced ERK phosphorylation in HSC. The phosphorylation of ERK induced by Ang II was significantly suppressed by Sal B and losartan (Figures 6(a) and 6(b)). However, there is little effect of Ang II and Sal B on total ERK levels. c-Jun was also activated by Ang II (Figures 6(c) and 6(d)), treatment with Sal B or losartan significantly inhibited c-Jun activation. 

### 3.7. Sal B Inhibited Ang II-Induced AT1R Upregulation

Since Ang II-induced HSC activation is mainly mediated by AT1R [12–14]. To further elucidate whether AT1R is involved in the inhibition of Sal B on Ang II-induced HSC activation, we examined AT1R levels in both HSC-T6 cells and primary rat HSC by western blot. After 24 h incubation, AT1R level was increased by Ang II (Figures 6(e), 6(f), 6(g), and 6(h)). Both Sal B and losartan treatment significantly prevented the increase of AT1R, indicating that down-regulating Ang II related signaling pathway is an important mechanism of Sal B effects on liver fibrosis.

## 4. Discussion

In the present study, we investigated the effects of Sal B on DMN-induced liver fibrosis in rats* in vivo *and on Ang II stimulated HSC activation *in vitro.* Firstly, with perindopril as control, which is one of ACEI and can inhibit Ang-II production, our results showed that Sal B and perindopril could attenuate DMN-induced rat liver fibrosis and down-regulate AT1R expression and ERK phosphorylation in rat liver. Secondly, with losartan as a control drug, which is one of angiotensin receptor blockers and can impair AT1R function, we found that Sal B and losartan could inhibit Ang II-induced type I collagen, TGF-*β*, *α*-SMA expression, and so forth in HSC *in vitro*. To elucidate the action mechanisms of Sal B related to Ang-II signaling, we observe main molecules involved in Ang II signal transduction. The results showed that Sal B reduced AT1R expression and ERK activation in fibrotic liver and inhibited AT1R expression and ERK and c-Jun phosphorylation in Ang II-stimulated HSC-T6 cells and primary rat HSC. These data indicate that Sal B could attenuate rat hepatic fibrosis via down-regulating Ang II signaling in HSC activation. 

In our previous studies, we found that Sal B prevent liver fibrosis in DMN-induced model and can inhibit TGF-*β*1 and its receptor expression, indicating that Sal B's action mechanism is associated with inhibition of TGF-*β*/Smads signaling [30, 35]. In the study, we confirmed that Sal B could promote the reversion of established liver fibrosis in DMN induced model, demonstrated by the decreased levels of *α*-SMA, Col-I, and hydroxyproline in Sal B treatment group, and Sal B had a similar effect on liver fibrosis as perindopril, which can reduce Ang-II production and liver or kidney fibrosis. In addition, Sal B reduced AT1R expression and ERK phosphorylation in fibrotic liver in the study. Therefore, we presumed that Ang II signaling pathway was another important target for Sal B antifibrotic action.

To further verify the effect of Sal B on Ang II signaling, we designed an experiment *in vitro*, in which both rat primary HSC and HSC-T6 cell line were applied [36]. HSC is a major source of ECM and its activation is an important step in hepatic fibrosis, while Ang II could increase HSC proliferation and induce its Col-I synthesis [34, 37, 38]. In the experiment, Ang II stimulated both primarily HSC and T6 cell line activation, demonstrated by the significant increase of Col-I and *α*-SMA expression in Ang-II treated cells. Ang II promotes liver fibrogenesis by its intracellular pathway as well as by regulating profibrotic cytokine expression such as TGF-*β*. TGF-*β* is one of the most potent profibrogenic cytokines known for activated HSC. Ang II could increase mRNA levels of all TGF-*β* isoforms in rat HSC [39]. In our previous study, we found that Sal B could inhibit TGF-*β*1 expressions and its receptor in fibrotic livers [14] and inhibit MAPK function in activated rat HSC [40]. In the present study, we found that Sal B could repress the increase of all TGF-*β* isoforms mRNA levels induced by Ang II. This result indicates that the inhibition of TGF-*β* production stimulated by Ang-II involves in Sal B action mechanism on liver fibrosis.

Ang II mainly activates HSC via the cell surface receptor, AT1R, a G protein coupled receptor. Once Ang II binds with AT1R, the intracellular signaling mediators and transcriptional factors would be activated, and then Ang II exerts its biological functions. Mitogen-activated protein kinases (MAPK) are important intracellular signaling mediators for Ang II signaling [41–43]. Previous study has documented that Ang II elicits a rapid and robust phosphorylation of ERK1/2 in HSC [12]. ERK1/2 can directly phosphorylate a set of transcription factors including c-Jun and c-Myc [44]. c-Jun is a subset of AP1, which can stimulate transcription of a wide variety of genes involved in hepatic fibrosis, such as Col-I and TGF*β*1 [45]. However, this effect can be inhibited or blocked by AT1R antagonist. Mice lacking AT1R do not develop chronic liver injury [13]. In the study, Ang II stimulated HSC activation and a fast phosphorylation of ERK1/2. Pretreatment with Sal B and losartan, an AT1R blocker, could inhibit AT1R expression, ERK and c-Jun phosphorylation in HSC activated by Ang II. In our experiment, incubation with Sal B could not only inhibit Ang II-induced fast phosphorylation of ERK, also down-regulate suggesting Sal B can act on multiple targets of the renin-angiotensin system in HSC. The data suggest that the down-regulation of Ang-II intracellular signal transduction is an important action mechanism of Sal B against HSC activation and liver fibrosis. 

In summary, Sal B, the active ingredient of *Salvia miltiorrhiza*, attenuated animal liver fibrosis and inhibited Ang II-induced HSC activation. The down-regulation of Ang-II signaling such as decreasing AT1R expression and ERK and c-Jun phosphorylation, and the inhibition of TGF-*β* expression stimulated by Ang-II is important action mechanism for Sal B against HSC activation and liver fibrosis. Our current works approve that the effect of Sal B relates to Ang II signal transduction in HSC activation and may provide a new sight to understand the anti-hepatic fibrosis effect of Sal B. 

## Figures and Tables

**Table 1:** Primer sequences used in this study.

Gene	Forward (5′–3′)	Reverse (5′–3′)
Col-I*α*	AGAGCATGACCGATGGATTC	CCTTCTTGAGGTTGCCAGTC
TGF*β*1	AAGCAGTGCCAGAACCCCCA	CGTGTTGCTCCACAGTTGACTTGA
TGF*β*2	TGTGGGTACCTTGATGCCATCCCG	GTCGAAGGAGAGCCATTCCCCCT
TGF*β*3	CTCCCGATGGCGAAAGGCCG	CCTTCACCTGACCACTCTGCCCT

## Glossary

*α*-SMA: Alpha-smooth muscle actin
ACEI: Angiotensin-converting enzyme inhibitors
Ang II: Angiotensin II
AP-1: Activating protein-1
AT1R: Ang II receptor type 1
DMN: Dimethylnitrosamine
FBS: Fetal bovine serum
HSC: Hepatic stellate cells
MAPK: Mitogen-activated protein kinase
NF-*κ*B: Nuclear factor *κ*B
RAS: Rennin-angiotensin system
Sal B: Salvianolic acid B
TCM: Traditional Chinese Medicine
TGF-*β*: Transforming growth factor beta.
